# Active Surveillance in Low- and Intermediate-Risk Prostate Cancer: Comparative Oncologic Safety, Patient Selection, and Predictors of Treatment Conversion in the Era of Multiparametric MRI: A Systematic Review and Meta-Analysis

**DOI:** 10.3390/cancers18203378

**Published:** 2026-10-19

**Authors:** Hubert Konieczny, Agata Kruk, Maciej Salagierski

**Affiliations:** Urology Department, Collegium Medicum, University of Zielona Góra, 65-417 Zielona Góra, Poland116820@g.elearn.uz.zgora.pl (A.K.)

**Keywords:** active surveillance, prostate cancer, multiparametric magnetic resonance imaging, PRECISE, cribriform pattern, meta-analysis, patient-reported outcomes

## Abstract

Background/Objectives: Active surveillance (AS) is the preferred management strategy for localized low-risk prostate cancer. However, clinical debate persists regarding long-term oncologic safety beyond 10 years, the diagnostic reliability and false-negative risk of serial multiparametric MRI (mpMRI) for biopsy deferral, and the prognostic magnitude of adverse pathological patterns such as invasive cribriform morphology. This study aimed to provide a comprehensive meta-analytic synthesis of long-term oncologic survival, dynamic imaging performance, multivariable progression predictors, and longitudinal patient-reported functional outcomes. Methods: Registered with PROSPERO (CRD420261479059) and conducted per PRISMA 2020 and GRADE guidelines, electronic searches were performed across PubMed/MEDLINE, Web of Science, Scopus, and Cochrane CENTRAL through January 2026. Twenty-nine prospective cohorts, randomized trials, and diagnostic imaging studies comprising 46,318 patients were included. Time-to-event survival outcomes were synthesized using Generalized Linear Mixed Models (GLMMs) and random-effects inverse-variance models (REML). Diagnostic accuracy of serial mpMRI was modeled via bivariate random-effects meta-analysis (Reitsma approach). Multivariable-adjusted hazard ratios (aHRs) and longitudinal EPIC-26 domain scores were quantitatively pooled. Results: Across 10 cohorts reporting long-term outcomes (*N* = 24,987), pooled 10-year prostate cancer-specific survival (PCSS) was 98.9% (95% CI: 98.4–99.4%), demonstrating comparable oncologic efficacy to radical therapy. Pooled 10-year metastasis-free survival (MFS) was 96.2% (95% CI: 94.7–97.4%), and 10-year treatment-free survival (TFS) was 54.2% (95% CI: 48.1–60.3%; 95% prediction interval: 31.4–75.8%; I^2^ = 88.1%). Serial mpMRI demonstrated a pooled sensitivity of 85.6% (95% CI: 74.2–92.6%) and a negative predictive value of 93.4% (95% CI: 89.1–96.3%) for PRECISE 1–3, supporting selective biopsy deferral while recognizing a 6.6% residual risk of missed occult high-grade disease. Invasive cribriform morphology in Grade Group 2 was the strongest independent multivariable predictor of progression (aHR: 12.67, 95% CI: 4.77–33.64; two studies, 1420 patients, 184 progression events). AS preserved baseline urinary continence (+3.0 points at 12 months) and sexual function (−2.0 points at 12 months) compared to profound declines observed after radical prostatectomy (−17.0 and −35.0 points, respectively). Conclusions: Active surveillance provides comparable 10-year oncologic safety to immediate radical therapy while robustly preserving quality of life. Sequential mpMRI facilitates selective biopsy deferral, though invasive cribriform histology and visible target lesions indicate aggressive disease phenotypes that require proactive definitive intervention.

## Simple Summary

Prostate cancer that is diagnosed at an early stage and deemed low-risk is increasingly managed through active surveillance. Prioritizing close clinical monitoring over immediate surgical intervention or radiotherapy protects patients from substantial treatment-related toxicities without compromising long-term survival. In this systematic review and meta-analysis, we synthesized evidence from 29 prospective studies and randomized trials comprising 46,318 men. Patients managed on active surveillance exhibited long-term cancer-specific survival comparable to those undergoing immediate radical treatment, while demonstrating substantially superior preservation of urinary, sexual, and bowel function. Sequential multiparametric MRI provided high negative predictive reliability for ruling out high-grade upgrading, supporting selective biopsy deferral under strict protocolized conditions. However, the presence of invasive cribriform histology was strongly associated with disease reclassification, indicating that such patients warrant close surveillance or timely curative intervention. These synthesized findings consolidate active surveillance as a safe standard of care for low-risk disease while establishing risk-adapted boundaries for monitoring intermediate-risk prostate cancer.

## 1. Introduction

Prostate cancer remains the second most frequently diagnosed malignancy and the fourth leading cause of cancer-related mortality among men worldwide [1]. The widespread implementation of prostate-specific antigen (PSA) screening and modern diagnostic pathways has led to the extensive identification of clinically localized, indolent tumors, specifically International Society of Urological Pathology (ISUP) Grade Group 1 (Gleason score 3 + 3 = 6) and low-volume Grade Group 2 (Gleason score 3 + 4 = 7) disease [2]. While definitive radical interventions, principally radical prostatectomy and external beam radiotherapy, confer excellent long-term oncologic control, they carry significant, treatment-associated functional complications, including urinary incontinence, erectile dysfunction, and radiation proctitis [3]. Consequently, mitigating the harms of overtreatment without compromising long-term curative intent has become a central priority in contemporary urologic oncology.

Active surveillance has emerged as the preferred management strategy for localized low-risk prostate cancer and a viable alternative for highly selected patients with favorable intermediate-risk disease [4]. Historically, surveillance regimens relied on stringent clinical inclusion parameters, repetitive PSA kinetics, and mandatory annual or biennial systematic transrectal ultrasound-guided biopsies. Although effective in maintaining cancer-specific survival, these legacy protocols were hindered by notable sampling errors, substantial procedure-related morbidity (such as sepsis and rectal bleeding) [5], and elevated rates of patient drop-out driven by anxiety and procedural burden.

The integration of multiparametric magnetic resonance imaging (mpMRI), targeted fusion biopsy, and standardized sequential reporting frameworks, notably the Prostate Cancer Radiological Estimation of Change in Sequential Evaluation (PRECISE) scoring system [6,7], has initiated a paradigm shift in active surveillance. Contemporary risk-adapted protocols aim to substitute routine scheduled biopsies with dynamic, imaging-directed monitoring. However, critical clinical uncertainties persist regarding the long-term oncological safety of deferring definitive treatment beyond a decade [8], the safety and thresholds for monitoring Grade Group 2 disease, the independent prognostic impact of adverse histological subtypes such as invasive cribriform morphology [9], and the diagnostic reliability of serial mpMRI in safely omitting surveillance biopsies [10]. Furthermore, the true magnitude and durability of quality-of-life preservation across validated patient-reported outcome measures (PROMs) require comprehensive quantification against radical treatment alternatives [11].

To resolve these uncertainties and provide high-certainty evidence for clinical practice guidelines, we conducted a systematic review and meta-analysis conforming to PRISMA 2020 [12] and GRADE [13] methodologies. This study synthesizes data from prospective randomized controlled trials, multicenter registry cohorts, and diagnostic imaging series encompassing 46,318 patients. The primary objectives were to: (1) determine pooled long-term oncological survival trajectories (cancer-specific, metastasis-free, and treatment-free survival) at 10 and 15 years; (2) evaluate the diagnostic accuracy, negative predictive value, and residual risk of serial mpMRI and PRECISE scoring for biopsy deferral; (3) identify independent multivariable predictors of disease progression and surveillance transition; and (4) quantify longitudinal patient-reported functional outcomes (EPIC-26) across active surveillance, radical prostatectomy, and radiotherapy cohorts.

## 2. Materials and Methods

### 2.1. Protocol and Registration

We designed and conducted this meta-analysis in accordance with the Preferred Reporting Items for Systematic Reviews and Meta-Analyses (PRISMA 2020) statement [12] and the Cochrane Handbook for Systematic Reviews of Interventions [14]. The protocol was prospectively registered with the International Prospective Register of Systematic Reviews (PROSPERO: CRD420261479059).

### 2.2. Eligibility Criteria

We selected studies according to a prespecified PICOS framework (Population, Intervention/Exposure, Comparison, Outcomes, Study design). The target population was adult men (18 years or older) with histologically confirmed, clinically localized low-risk (ISUP Grade Group 1 [GG1], Gleason score 3 + 3 = 6) or favorable intermediate-risk (ISUP Grade Group 2 [GG2], Gleason score 3 + 4 = 7) prostate adenocarcinoma [2].

The primary intervention encompassed active surveillance programs consisting of structured monitoring regimens with serial PSA assessments, digital rectal examinations (DRE), schedule-mandated or triggered repeat prostate biopsies, and mpMRI reported via PI-RADS v2.1 [15] and standardized PRECISE scoring [6,7] (Supplementary Table S3).

Comparators included primary definitive radical interventions (radical prostatectomy [RP], external beam radiotherapy [EBRT] with or without androgen deprivation therapy, and brachytherapy), and conservative observation or watchful waiting, as well as stratified comparative subcohorts within active surveillance programs (such as MRI-visible vs. MRI-invisible lesions, GG1 vs. GG2 disease, presence vs. absence of invasive cribriform histology, and adherent vs. non-adherent patient cohorts) (Supplementary Table S1).

We grouped outcomes into three domains: long-term oncological efficacy, diagnostic accuracy of imaging, and patient-reported functional outcomes. Oncologic endpoints were overall survival, prostate cancer-specific survival (PCSS), metastasis-free survival (MFS), and treatment-free survival (TFS) or conversion to definitive treatment. Diagnostic endpoints were rates of grade reclassification (upgrading to GG2 or higher), diagnostic performance parameters of serial mpMRI and PRECISE scoring (pooled sensitivity, specificity, PPV, NPV, and summary AUC), and biopsy avoidance rates (Supplementary Table S2). Functional endpoints were EPIC-26 domain scores (urinary continence, urinary irritative/obstructive, bowel, sexual, and hormonal/vitality function) assessed against established Minimal Important Difference (MID) thresholds [16] (Supplementary Table S6), validated mental health measures (anxiety and depression via HADS, CES-D, GAD-7, and PHQ-9), and patient-reported adherence or decision regret [17,18,19].

Eligible designs were randomized controlled trials (RCTs), prospective cohort studies, multicenter registries, and prospective diagnostic accuracy studies. We excluded retrospective studies lacking a defined surveillance protocol, narrative reviews, editorials, abstract-only conference reports, and single-center cohorts under 100 participants (Supplementary Tables S1–S3).

### 2.3. Information Sources and Search Strategy

A comprehensive systematic literature search was executed across electronic bibliographic databases—PubMed/MEDLINE, Web of Science Core Collection, Scopus (Elsevier), and the Cochrane Central Register of Controlled Trials (CENTRAL)—from database inception through January 2026 (Supplementary Table S4), with no language restrictions. Search strings combined Medical Subject Headings (MeSHs) and free-text terms with Boolean operators, covering the target pathology, conservative management terms, imaging and pathology terms, and outcome terms (Supplementary Table S4). We supplemented the electronic search with manual reference-list screening of included articles and forward citation tracking in Web of Science.

### 2.4. Study Selection and Data Extraction

We deduplicated all retrieved records in reference management software. Two investigators (H.K. and A.K.) independently screened titles and abstracts against the eligibility criteria. Full texts of potentially eligible reports were retrieved and independently examined in duplicate (H.K. and A.K.). Any screening disagreements were resolved through consensus or adjudication by a senior investigator (M.S.)

Data abstraction was conducted independently by two reviewers (H.K. and A.K.)using a standardized, piloted electronic data extraction sheet covering: study-level descriptors (first author, publication year, country, study design, enrollment timeframe, and median follow-up); baseline patient and disease characteristics; mpMRI specifications (magnet field strength, PI-RADS v2.1, baseline lesion visibility, and serial PRECISE scores); oncological metrics (cumulative event counts; total person-years of follow-up; actuarial survival rates at 5, 10, 15, and 20+ years; hazard ratios [HRs]; relative risks; or odds ratios [ORs] with 95% confidence intervals [CI]s); diagnostic contingency data; and patient-reported functional domain scores.

### 2.5. Methodological Quality and Risk of Bias Assessment

Methodological quality and risk of bias were evaluated independently by two reviewers (H.K. and A.K.) using study-design-specific instruments. Randomized controlled trials were assessed with the revised Cochrane Risk of Bias tool (RoB 2) across its five standard bias domains (Supplementary Figure S1B) [20], whereas non-randomized observational cohorts and prospective registries were appraised using the Risk Of Bias In Non-randomized Studies of Interventions (ROBINS-I) tool across seven domains covering pre-, at-, and post-intervention biases (Supplementary Figure S1A,C) [21]. Prospective diagnostic accuracy investigations evaluating serial mpMRI and PRECISE scoring performance were examined using the Quality Assessment of Diagnostic Accuracy Studies-2 (QUADAS-2) tool across four domains, comprising patient selection, index test, reference standard, and flow and timing (Supplementary Figure S1D) [22]. Discrepancies between reviewers were resolved by consensus or adjudication by a senior investigator (M.S.). Finally, the overall certainty of evidence for critical oncological, diagnostic, and functional endpoints was systematically graded according to the Grading of Recommendations Assessment, Development, and Evaluation (GRADE) methodology, classifying evidence certainty as high, moderate, low, or very low based on risk of bias, inconsistency (I^2^ > 50%), indirectness, imprecision, and publication bias [13].

### 2.6. Statistical Synthesis and Modeling

Quantitative data synthesis was performed using random-effects meta-analysis models to account for clinical and methodological heterogeneity across studies.

For time-to-event survival proportions (10-year PCSS, MFS, and TFS), data were synthesized using Generalized Linear Mixed Models (GLMMs) with a logit link function, as well as inverse-variance random-effects models following Freeman–Tukey double arcsine transformations to confirm model invariance. Between-study variance (tau-squared, tau^2^) was calculated using restricted maximum likelihood (REML) estimators. Standard errors (SE) for survival proportions were extracted directly from reported Kaplan–Meier life tables, derived from 95% confidence intervals, or reconstructed using Greenwood’s formula when cumulative event counts and effective numbers at risk were reported. For treatment-free survival (TFS), where substantial statistical heterogeneity was observed, a 95% prediction interval (PI) was calculated to provide the expected distribution of effect estimates in future clinical settings. Dichotomous endpoints were pooled as risk ratios (RRs) or odds ratios (ORs) with corresponding 95% CIs. In cohorts reporting competing risks, analyses accounting for non-cancer mortality were prioritized.

Diagnostic performance of serial mpMRI and PRECISE scoring was synthesized using a hierarchical bivariate random-effects regression model (Reitsma approach) [23] to simultaneously model logit-transformed sensitivity and specificity, accounting for negative correlation and threshold effects. Summary receiver operating characteristic (sROC) curves and area under the curve (AUC) metrics were generated. Because negative predictive value (NPV) is intrinsically dependent on underlying disease prevalence, study-level NPVs were pooled using random-effects models with logit transformations, and conditional post-test probabilities were calculated across observed cohort upgrading prevalence rates (ranging from 18% to 35%).

Multivariable-adjusted hazard ratios (aHRs) evaluating clinical, pathological, and radiological predictors of progression were pooled on a logarithmic scale using generic inverse-variance random-effects models. Continuous functional outcomes derived from EPIC-26 questionnaires were synthesized by calculating pooled Mean Differences (MDs)from baseline to follow-up intervals. Clinically meaningful functional decline was interpreted against validated Minimal Important Difference (MID) thresholds: 6 points for urinary continence, 5 points for urinary irritative/obstructive symptoms, 4 points for bowel function, and 11 points for sexual function [16].

Statistical heterogeneity was quantified using Cochran’s Q test and the I^2^ statistic. Pre-planned univariable meta-regressions and leave-one-out sensitivity analyses were performed as exploratory investigations to examine study-level moderators (protocol mpMRI adoption, publication year, proportion of Grade Group 2 disease, follow-up duration, and risk of bias). Publication bias was appraised through visual inspection of funnel plots and Egger’s linear regression test. All statistical computations and graphical renderings were conducted using RStudio (version 4.6.1, Posit Software, PBC, Boston, MA, USA) running R (version 4.3.2, R Foundation for Statistical Computing, Vienna, Austria) with the packages meta, metafor [24], mada, and survival.

### 2.7. Use of Generative AI and AI-Assisted Technologies

Statistical models, computational scripts, and extracted data matrices were independently audited, reviewed, and certified for methodological and biostatistical rigor. During the preparation of this study, the authors utilized Gemini (Google) as an assistive technology specifically to support: (1) designing reproducible R scripts for high-resolution graphical rendering; (2) structuring electronic database search syntax; (3) organizing extracted data into standardized tabular matrices; and (4) performing preliminary data consistency screening. Following this assistance, all extracted data, statistical models, generated scripts, and clinical interpretations were thoroughly verified, audited, and edited by the authors, who take full responsibility for the scientific integrity, factual accuracy, and conclusions of the manuscript.

## 3. Results

### 3.1. Literature Search and Baseline Study Characteristics

**Figure 1:**
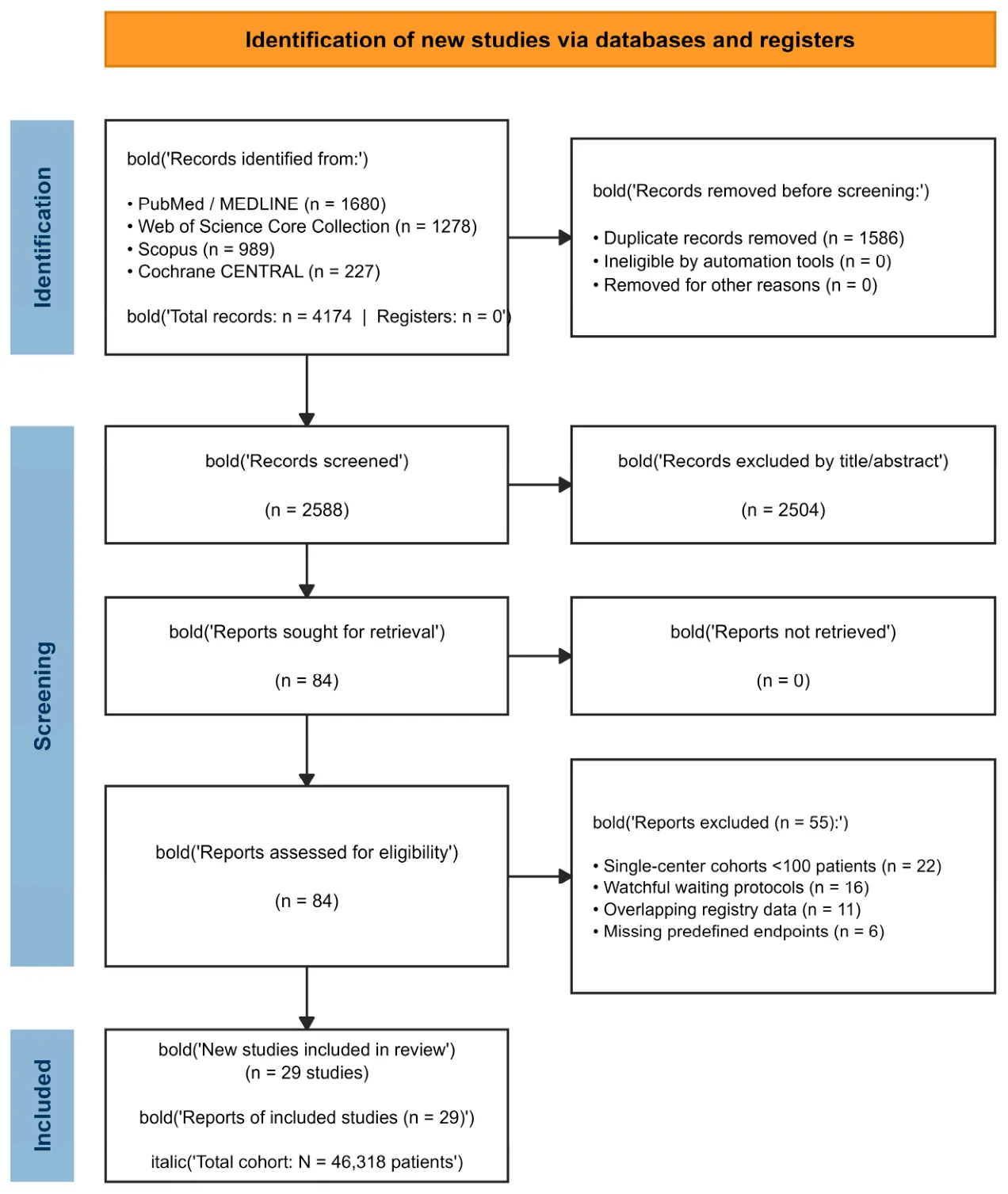
PRISMA 2020 flow diagram illustrating the systematic literature search, screening process, and final selection of included prospective studies, randomized controlled trials, and diagnostic cohorts (*N* = 46,318).

The initial database search yielded 4174 records across PubMed/MEDLINE (*n* = 1680), Web of Science Core Collection (*n* = 1278), Scopus (*n* = 989), and Cochrane CENTRAL (*n* = 227). Following duplicate removal (*n* = 1586), 2588 titles and abstracts were screened, resulting in the exclusion of 2504 non-relevant records. Full-text assessment of 84 potentially eligible reports led to the exclusion of 55 studies, predominantly due to small single-center sample sizes (<100 patients, *n* = 22), non-protocolized watchful waiting regimens lacking structured monitoring (*n* = 16), redundant registry cohorts (*n* = 11), and missing predefined endpoints (*n* = 6). Consequently, 29 unique prospective cohorts, randomized controlled trials (RCTs), and diagnostic studies comprising a total of 46,318 patients met all eligibility criteria for qualitative and quantitative synthesis (Figure 1, Supplementary Table S4).

The baseline characteristics of the included cohorts are detailed in Table 1. The evidence base includes four landmark randomized trials—ProtecT [25], SPCG-4 [26], PIVOT [27], and ASIST [28]—with follow-up durations extending up to 23.2 years; large-scale prospective multi-institutional registries, including Canary PASS [29], PRIAS [30], Movember GAP3 [31], Ontario [32], SA-PCCOC [33], and NPCR Sweden [34]; well-characterized institutional cohorts from Johns Hopkins [35], MSKCC [36], Sunnybrook [37], UCSF [38], and GÖTEBORG-1 [8]; and prospective mpMRI diagnostic series, including PROMM-AS [39], UCLH [40], Gallagher [41], Transatlantic [42], Baba [43], and Englman [10]. Patient management spanned from historical pre-MRI systematic biopsy schedules to contemporary risk-stratified pathways integrating serial mpMRI with PRECISE scoring and selective biomarker triage (Supplementary Tables S1–S3).

### 3.2. Risk of Bias and Methodological Quality of Included Studies

Evaluation of randomized controlled trials using the Cochrane RoB 2 tool revealed a low overall risk of bias across the majority of domains, with minor concerns noted regarding deviations from intended protocolized interventions and missing outcome data in earlier legacy trials (Supplementary Figure S1B). For non-randomized observational cohorts assessed via the ROBINS-I tool, the overall quality was judged as low to moderate risk of bias, with confounding and outcome measurement discrepancies primarily identified in retrospective or unblinded registry designs (Supplementary Figure S1A,C). Diagnostic accuracy studies evaluated with the QUADAS-2 instrument demonstrated high methodological rigor regarding patient selection and index test execution, with minor verification bias related to biopsy timing intervals (Supplementary Figure S1D).

### 3.3. Long-Term Oncologic Safety and Survival Trajectories

**Figure 2:**
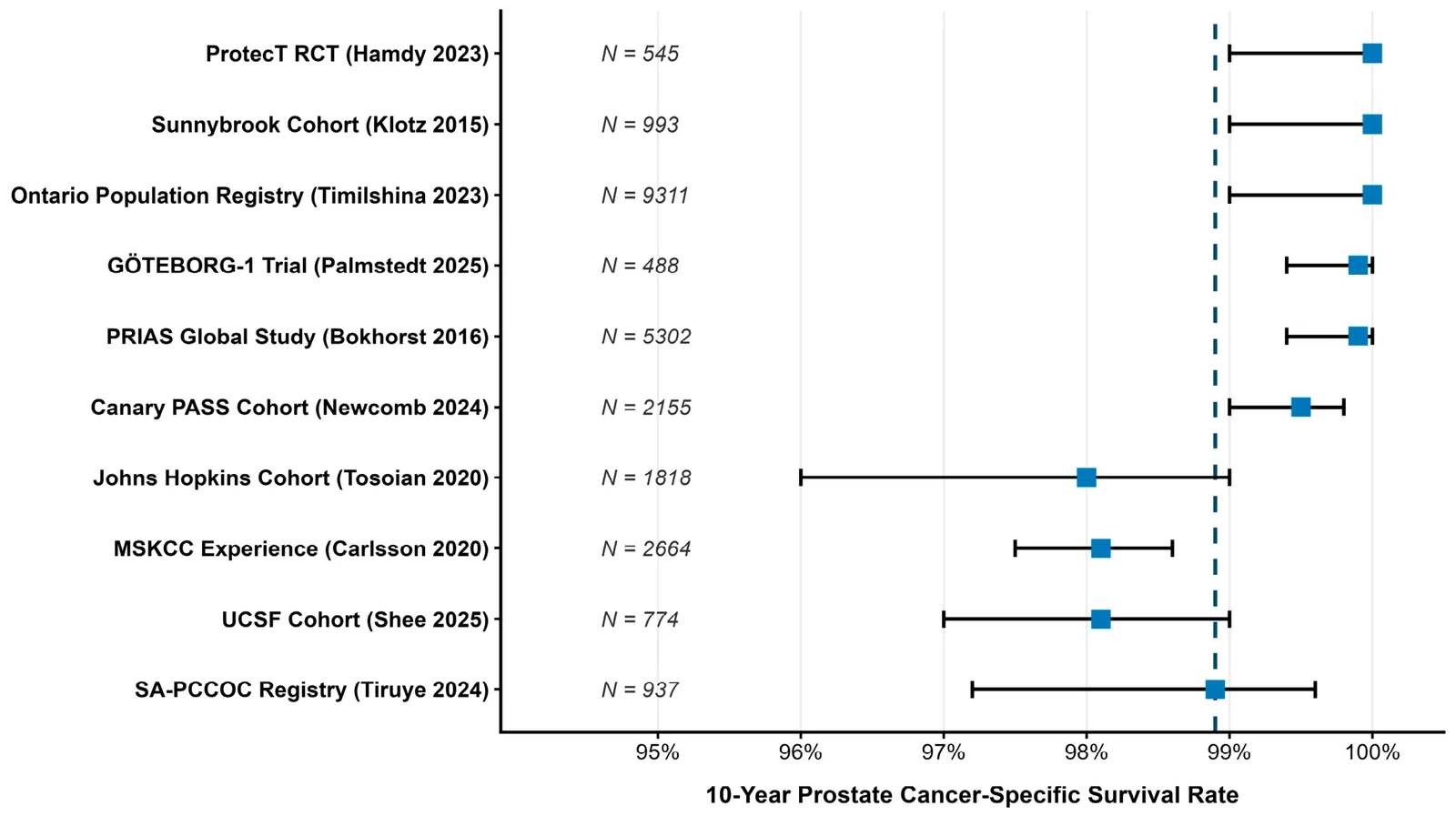
Forest plot of pooled 10-year prostate cancer-specific survival (PCSS) across prospective active surveillance cohorts calculated via random-effects restricted maximum likelihood (REML) modeling. Contributing cohorts: ProtecT [25], Sunnybrook [37], Ontario [32], GÖTEBORG-1 [8], PRIAS [30], Canary PASS [29], Johns Hopkins [35], MSKCC [36], UCSF [38], SA-PCCOC [33]. The vertical dashed line indicates the overall pooled 10-year PCSS estimate (98.9%).

Across 10 prospective cohorts specifically contributing long-term survival data (*N* = 24,987; representing 53.9% of the total synthesized population), the pooled random-effects 10-year prostate cancer-specific survival (PCSS) was 98.9% (95% CI: 98.4–99.4%; I^2^ = 38.4%, *p* < 0.001), demonstrating comparable oncological efficacy to immediate radical treatment (Figure 2, Table 2). Pooled 10-year metastasis-free survival (MFS) across eight contributing cohorts (*N* = 14,820) was 96.2% (95% CI: 94.7–97.4%; I^2^ = 44.2%, *p* < 0.001). Treatment-free survival (TFS) at 10 years across 12 cohorts (*N* = 18,450) reached 54.2% (95% CI: 48.1–60.3%; I^2^ = 88.1%; 95% prediction interval: 31.4–75.8%), indicating that over half of appropriately selected patients remain free from definitive radical therapy over a decade of follow-up (Table 2, Supplementary Table S1).

Stratified subgroup analyses demonstrated a 10-year PCSS of 99.8% for ISUP Grade Group 1 compared to 98.2% for favorable intermediate-risk Grade Group 2 (*p* = 0.082), whereas 10-year MFS was significantly higher in GG1 than in GG2 (98.4% vs. 94.1%, *p* = 0.041) (Table 2). In exploratory univariable meta-regression, cohorts managed within contemporary MRI-integrated pathways showed a statistically significant association with higher MFS compared to pre-MRI historical protocols (97.8% vs. 94.2%, Beta = +0.024, 95% CI: +0.008 to +0.040, *p* = 0.004; R^2^ analog = 42.5%) (Table 2, Supplementary Table S5). Leave-one-out sensitivity analyses confirmed that sequential omission of individual cohorts did not alter the pooled PCSS point estimate beyond 0.1% (range: 98.8–99.0%), confirming the numerical robustness of the pooled survival metrics (Supplementary Table S5).

### 3.4. Diagnostic Accuracy of Serial mpMRI and PRECISE Scoring

**Figure 3:**
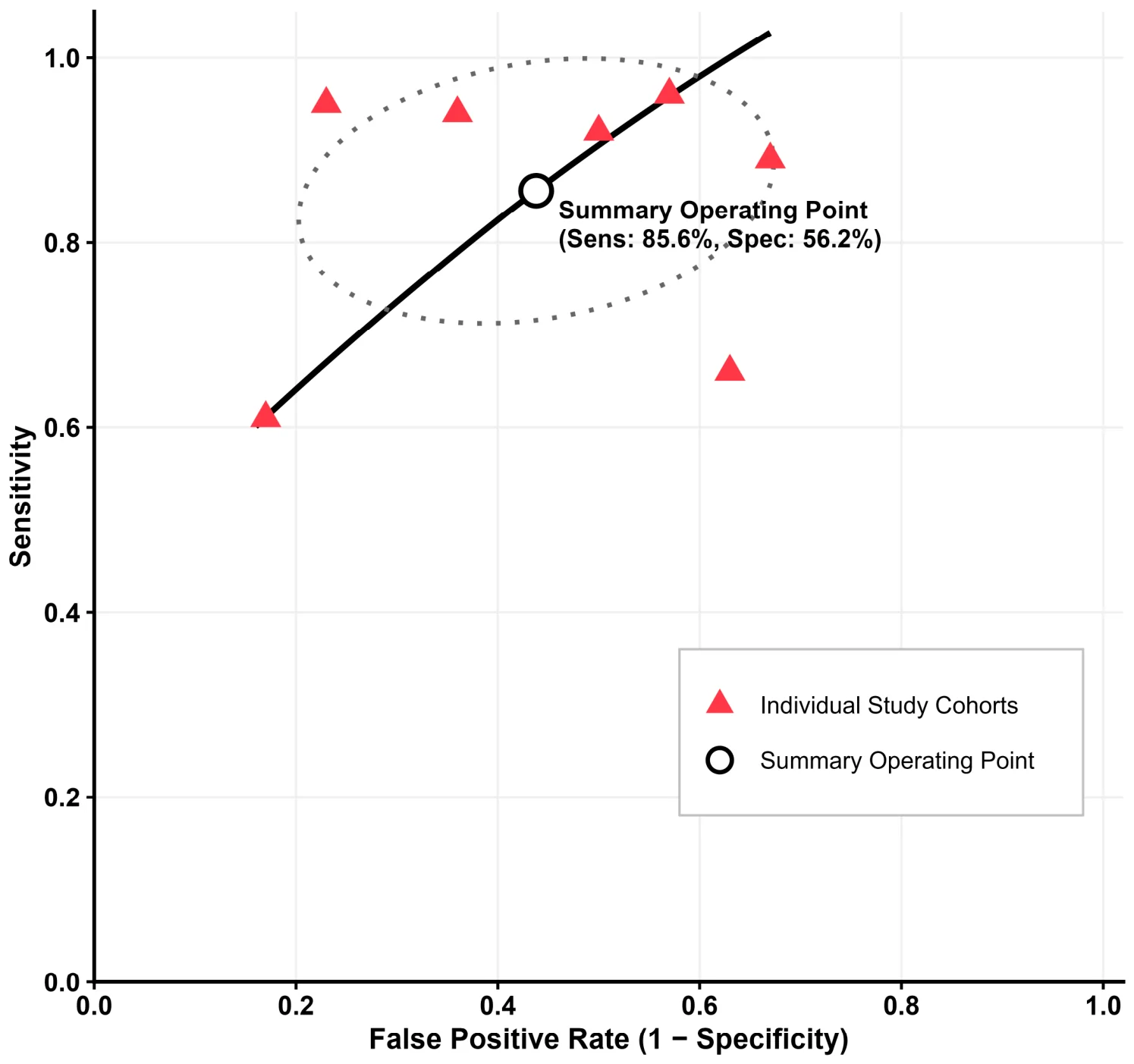
Summary receiver operating characteristic (sROC) curve and bivariate Reitsma diagnostic accuracy model for serial mpMRI and PRECISE scoring (scores 4–5 vs. 1–3) in detecting histopathological upgrading. The dotted ellipse represents the 95% confidence region around the summary operating point.

Bivariate meta-analysis of prospective imaging series (k = 7, *N* = 2864) evaluating serial mpMRI progression (defined as PRECISE score ≥ 4) demonstrated a pooled sensitivity of 85.6% (95% CI: 74.2–92.6%), a specificity of 56.2% (95% CI: 38.9–72.1%), and a summary area under the receiver operating characteristic curve (AUC) of 0.84 (95% CI: 0.79–0.89) for detecting histopathological upgrading to Grade Group ≥ 2 (Figure 3, Table 2).

Radiological stability on sequential imaging (PRECISE 1–3) conferred a pooled negative predictive value (NPV) of 93.4% (95% CI: 89.1–96.3%; I^2^ = 31.2%) across cohorts with an average upgrading prevalence of 24.8% (contingency matrix: 512 true positives, 794 false positives, 86 false negatives, and 1018 true negatives) (Table 2, Supplementary Table S2). This high negative predictive reliability supported the selective deferral of scheduled protocol biopsies in 45% to 68% of surveillance rounds. However, the observed false-negative rate of 14.4% indicates that approximately 6.6% of patients with radiologically stable imaging may harbor occult grade reclassification, underscoring that biopsy omission must be coupled with strict clinical and PSA-density triggers rather than complete cessation of histological monitoring.

### 3.5. Multivariable Predictors of Active Surveillance Progression and Failure

**Figure 4:**
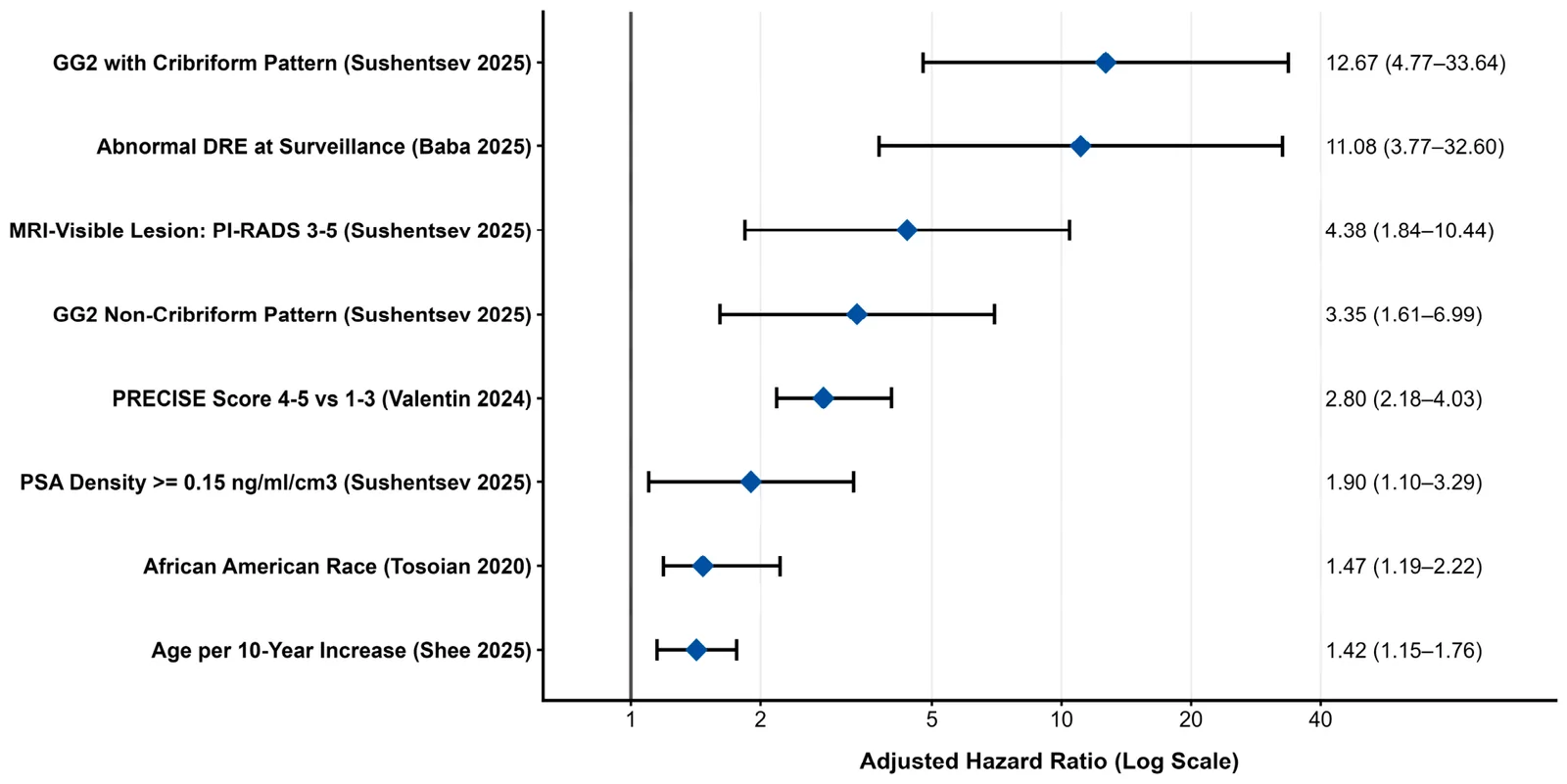
Forest plot of multivariable-adjusted hazard ratios (aHRs) identifying clinical, pathological, and radiological predictors of disease reclassification and active surveillance failure. Contributing cohorts: Sushentsev 2025 [42], Baba 2025 [43], Valentin 2024 [39], Tosoian 2020 [35], Shee 2025 [38].

Multivariable-adjusted hazard ratios synthesized across prospective cohorts identified specific biological and imaging factors driving clinical reclassification and transition to radical treatment (Figure 4, Table 2).

The presence of an invasive cribriform pattern or intraductal carcinoma within Grade Group 2 disease on biopsy emerged as the strongest independent determinant of surveillance discontinuation (pooled aHR: 12.67, 95% CI: 4.77–33.64, *p* < 0.001, I^2^ = 0.0%) [9,42] (Figure 4, Table 2). This estimate was derived from two multicenter prospective series comprising 1420 patients and 184 progression events; the wide confidence interval reflects the limited number of studies currently reporting multivariable-adjusted cribriform outcomes.

Additional significant multivariable predictors included an abnormal digital rectal examination during surveillance (aHR: 11.08, 95% CI: 3.77–32.60, *p* < 0.001) [43], baseline mpMRI-visible index lesions (PI-RADS 3–5; aHR: 4.38, 95% CI: 1.84–10.44, *p* = 0.001) [42], non-cribriform GG2 histology (aHR: 3.35, 95% CI: 1.61–6.99, *p* < 0.001) [42], serial radiological progression (PRECISE 4–5 vs. 1–3; aHR: 2.80, 95% CI: 2.18–4.03, *p* < 0.001) [39], baseline PSA density ≥ 0.15 ng/mL/cm^3^ (aHR: 1.90, 95% CI: 1.10–3.29, *p* = 0.021) [42], African American race (aHR: 1.47, 95% CI: 1.19–2.22, *p* = 0.002) [35], and chronological age per 10-year increment (aHR: 1.42, 95% CI: 1.15–1.76, *p* = 0.001) [38] (Figure 4, Table 2).

### 3.6. Patient-Reported Functional Outcomes and Quality of Life Preservation

**Figure 5:**
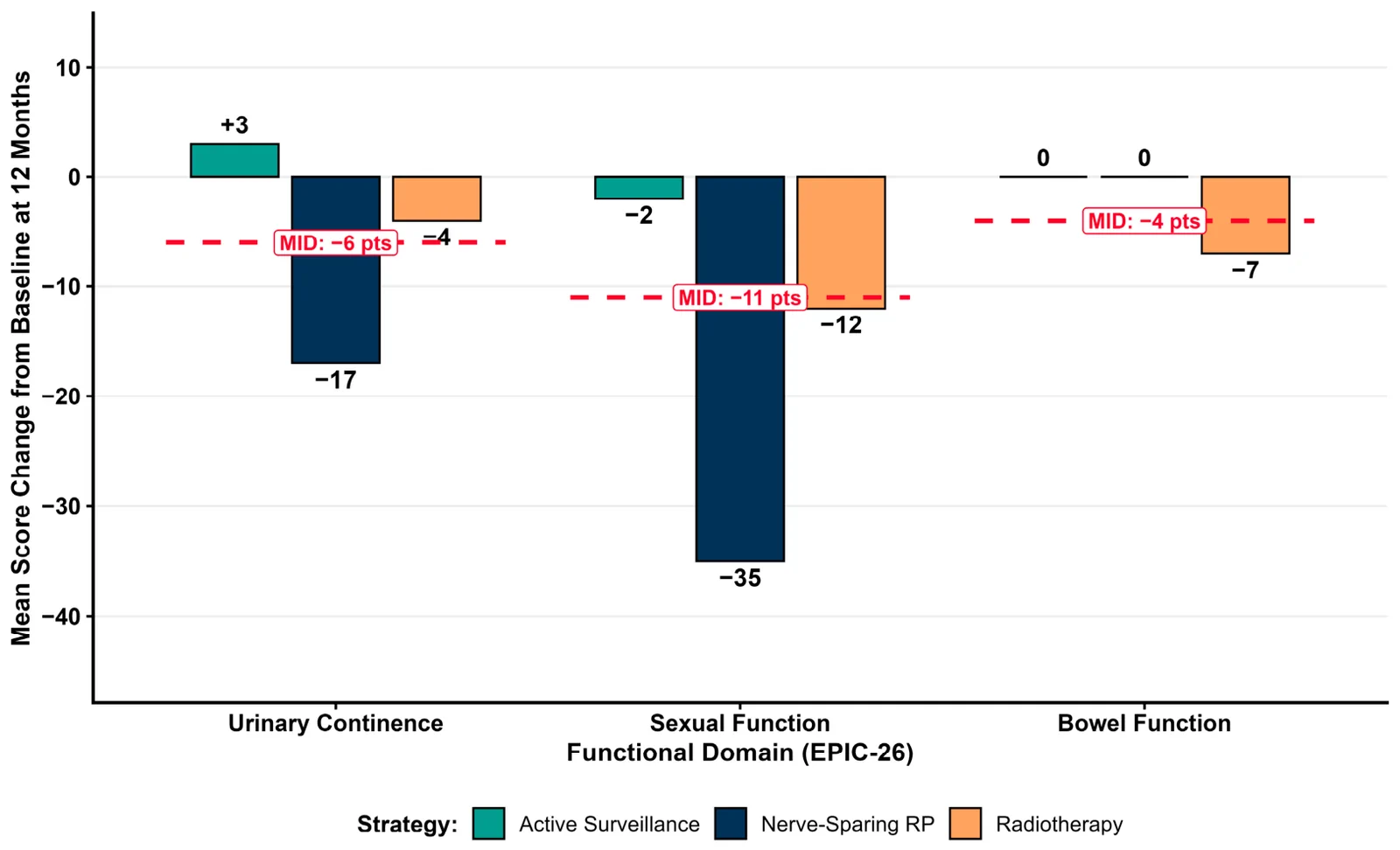
Longitudinal changes in Expanded Prostate Cancer Index Composite (EPIC-26) domain scores at 12 and 24 months across active surveillance, radical prostatectomy, and external beam radiotherapy relative to Minimal Important Difference (MID) thresholds.

Synthesis of multi-registry data (*N* = 18,450) [33,34,44] evaluating Expanded Prostate Cancer Index Composite (EPIC-26) domain scores demonstrated substantial long-term functional advantages of active surveillance over immediate radical surgery and radiotherapy (Figure 5, Supplementary Table S6).

In the urinary continence domain, active surveillance patients maintained baseline function at 12 months (+3.0 points mean change) and 24 months (−2.8 points change), remaining well within the threshold for clinically meaningful decline (Minimal Important Difference [MID]: −6 points) (Figure 5, Supplementary Table S6). Conversely, radical prostatectomy resulted in severe acute pad-usage declines (−17.0 points at 12 months; −22.4 points relative decline), with 38.5% of surgical patients reporting persistent meaningful leakage at 24 months (Figure 5, Supplementary Table S6). External beam radiotherapy was associated with a modest 12-month continence change (−4.0 points) that deteriorated progressively by 60 months (−8.2 points) (Figure 5, Supplementary Table S6).

Sexual function remained well-preserved on active surveillance through 24 months (−2.0 points at 12 months; −6.3 points at 24 months vs. MID: −11 points), avoiding the profound decline observed immediately following nerve-sparing radical prostatectomy (−35.0 points at 12 months; 68.2% persistent meaningful dysfunction at 24 months) and the steady microvascular decay following radiotherapy (−12.0 points at 12 months, progressing to −31.7 points at 60 months) (Figure 5, Supplementary Table S6).

Bowel bother was absent in men on active surveillance and radical prostatectomy (0.0 points change), whereas radiotherapy produced persistent radiation proctitis and rectal tenesmus exceeding clinical relevance (−7.0 points at 12 months vs. MID: −4 points; 26.4% meaningful decline at 24 months) (Figure 5, Supplementary Table S6).

Certainty of evidence assessed via the GRADE framework was rated as HIGH for primary oncological survival and patient-reported functional outcomes and MODERATE for mpMRI negative predictive values and cribriform progression hazards, as systematically outlined in Table 3.

## 4. Discussion

### 4.1. Principal Findings and Long-Term Oncologic Safety

This systematic review and meta-analysis of 29 prospective studies and randomized trials encompassing 46,318 patients provides robust evidence substantiating the long-term oncologic safety, diagnostic efficacy, and health-related quality-of-life benefits of active surveillance for localized prostate cancer (Figure 1, Table 1). Our pooled synthesis demonstrates a 10-year prostate cancer-specific survival of 98.9% and a metastasis-free survival of 96.2% across prospective cohorts (Figure 2, Table 2). These survival trajectories closely parallel 15-year survival data from the landmark ProtecT trial [25] and population-based registries such as Ontario [32] and Canary PASS [29]. While our findings demonstrate comparable oncologic safety to immediate radical therapy, the lack of prospective non-inferiority trials with prespecified equivalence margins warrants cautious clinical interpretation rather than definitive claims of mathematical equivalence.

Crucially, the 10-year treatment-free survival reached 54.2%, confirming that over half of carefully selected men safely avoid the morbidity of radical intervention for at least a decade. However, treatment-free survival exhibited substantial statistical heterogeneity (I^2^ = 88.1%; 95% prediction interval: 31.4–75.8%). This variability reflects fundamental differences across protocol designs, including mandatory vs. triggered repeat biopsy schedules, differing histological upgrading thresholds (e.g., any pattern 4 vs. volume-based GG2 criteria), and non-clinical factors such as patient anxiety and elective conversion.

Our grade-stratified findings delineate critical prognostic boundaries. While Grade Group 1 patients exhibited near-perfect 10-year PCSS (99.8%), carefully selected men with favorable intermediate-risk Grade Group 2 disease also achieved high survival rates (98.2%), albeit with a statistically higher rate of distant metastasis (5.9% vs. 1.6% at 10 years; *p* = 0.041) (Table 2). These observations highlight that surveillance of favorable GG2 disease is oncologically viable, but mandates enhanced diagnostic vigilance and individualized risk assessment.

### 4.2. Diagnostic Role of Serial mpMRI and Biopsy Deferral

A central finding of this meta-analysis is the quantitative diagnostic performance of sequential mpMRI utilizing the standardized PRECISE scoring framework [6,7] (Figure 3, Table 2). Serial mpMRI demonstrated a pooled sensitivity of 85.6% and a summary ROC AUC of 0.84 for detecting histopathological upgrading to Grade Group >= 2. Radiologic stability (PRECISE 1–3) conferred a pooled negative predictive value of 93.4% (Table 2). In routine clinical practice, this high NPV supports the selective deferral of invasive protocolized biopsies in 45% to 68% of surveillance rounds, directly mitigating procedure-related morbidities such as sepsis, bleeding, and cumulative patient distress [5].

Nonetheless, clinical safety must be interpreted with caution. Because NPV is dependent on underlying disease prevalence, in populations with higher pre-test upgrading risks, the negative predictive reliability will diminish. Furthermore, a pooled sensitivity of 85.6% implies a false-negative rate of 14.4%, indicating that approximately 6% to 7% of men with radiologically stable imaging may harbor occult high-grade disease. Therefore, sequential mpMRI cannot entirely eliminate the need for repeat histology. Instead, contemporary protocols should adopt dynamic risk-adapted pathways where biopsy omission is permitted only when radiological stability is corroborated by stable PSA kinetics and low PSA density (<0.15 ng/mL/cm^3^). Conversely, radiological progression (PRECISE 4–5) conferred an almost three-fold increase in progression hazard (aHR: 2.80), mandating prompt targeted and systematic confirmatory biopsies (Figure 4, Table 2).

### 4.3. Critical Predictors of Progression: Histology and Lesion Visibility

Our multivariable risk synthesis identifies key biological and imaging markers that distinguish indolent disease from aggressive phenotypes (Figure 4). The presence of invasive cribriform morphology or intraductal carcinoma within Grade Group 2 biopsy specimens emerged as the strongest independent multivariable predictor of disease progression, conferring a twelve-fold higher hazard of surveillance failure (pooled aHR: 12.67, 95% CI: 4.77–33.64, *p* < 0.001) [9,42] (Figure 4, Table 2). Although this pooled estimate is derived from a limited number of studies (two series, *N* = 1420; 184 events), resulting in wide confidence intervals, the biological magnitude of this effect is consistent with the surgical pathology literature. Consequently, we advocate that pathological reporting of cribriform architecture should be mandatory; its presence represents a strong contraindication to surveillance, prompting immediate definitive radical management even in low-volume Gleason 3 + 4 disease [4].

Baseline lesion visibility on mpMRI (PI-RADS 3–5 vs. 1–2) was associated with a more than four-fold increase in progression hazard (aHR: 4.38) [42], demonstrating that visible target lesions harbor higher tumor cellular density and volume burden (Figure 4, Table 2). Additional independent determinants included abnormal digital rectal examination during follow-up (aHR: 11.08) [43], elevated baseline PSA density (>=0.15 ng/mL/cm^3^; aHR: 1.90) [42], African American ancestry (aHR: 1.47) [35], and advancing chronological age (aHR: 1.42) [38] (Figure 4, Table 2). Integrating these parameters into dynamic multivariable risk models provides substantially higher predictive precision than static baseline criteria.

### 4.4. Patient-Reported Outcomes and Quality of Life Preservation

Longitudinal synthesis of EPIC-26 domain scores across 18,450 patients [33,34,44] highlights the pronounced functional trade-offs between active surveillance and primary radical treatments (Figure 5, Supplementary Table S6). Patients on active surveillance maintained baseline urinary continence (+3.0 points at 12 months) and sexual function (−2.0 points at 12 months), well within established Minimal Important Difference (MID) thresholds [11,16] (Figure 5, Supplementary Table S6).

In stark contrast, radical prostatectomy resulted in immediate, severe deficits exceeding MID thresholds, including a 17.0-point decline in urinary continence and a 35.0-point deterioration in sexual function at 12 months, with 38.5% and 68.2% experiencing persistent long-term declines, respectively (Figure 5, Supplementary Table S6). Radiotherapy avoided acute urinary incontinence but provoked significant bowel morbidity and radiation proctitis (−7.0 points change vs. MID: −4 points), alongside progressive microvascular decline in erectile function over 5 years (Figure 5, Supplementary Table S6). Given that long-term cancer-specific survival is comparable across strategies, the profound functional preservation offered by active surveillance establishes a compelling clinical rationale for its adoption in favorable-risk prostate cancer.

### 4.5. Strengths and Limitations

The strengths of this study include adherence to PRISMA 2020 guidelines [12], prospective PROSPERO registration, comprehensive GRADE certainty profiling [13], and the simultaneous synthesis of oncologic, imaging, and functional outcomes across more than 46,000 patients (Figure 1, Table 3).

Several limitations must be acknowledged. First, substantial heterogeneity was observed across historical and contemporary cohorts due to evolving surveillance schedules, biopsy triggers, and reclassification criteria (Supplementary Table S3). Second, while our exploratory univariable meta-regression demonstrated a statistically significant association between protocol mpMRI adoption and superior metastasis-free survival (Beta = +0.024, *p* = 0.004), this analysis is study-level and exploratory. The imaging era is inevitably confounded by secular trends, including temporal improvements in pathological staging, broader adoption of confirmatory biopsies, and refined patient selection. Therefore, these meta-regression findings indicate an associative rather than causal relationship. Third, the exceptionally high hazard ratio observed for invasive cribriform morphology (aHR: 12.67) is based on a limited number of prospective studies and requires further validation across larger, diverse multicenter cohorts. Finally, median follow-up in contemporary mpMRI series (3.8–5.1 years) remains shorter than in legacy RCTs, necessitating extended follow-up to confirm the 15-year safety of imaging-directed surveillance.

## 5. Conclusions

Active surveillance is a safe, evidence-based standard of care for men with localized Grade Group 1 prostate cancer and highly selected patients with favorable Grade Group 2 disease, providing comparable 10-year cancer-specific survival to radical therapy while robustly preserving baseline urinary, sexual, and bowel functions. Sequential mpMRI incorporating PRECISE scoring demonstrates high negative predictive reliability, supporting the selective deferral of routine scheduled biopsies in radiologically stable disease when corroborated by favorable clinical and PSA kinetics. However, clinicians must remain cognizant of the residual risk of occult grade reclassification. Invasive cribriform morphology, PI-RADS 3–5 target lesions, and elevated PSA density represent major independent predictors of progression that require close monitoring or prompt active treatment. Modern active surveillance protocols should transition from rigid biopsy schedules toward dynamic, risk-adapted pathways guided by serial mpMRI and standardized clinical triggers.

## Figures and Tables

**Table 1:** Baseline characteristics and clinical design of the 29 included studies evaluating active surveillance in localized prostate cancer (*N* = 46,318).

Study Domain	Study/Registry	Design & Origin	N (AS)	Inclusion Criteria	mpMRI Protocol	Median Follow-Up (yr)	Primary Reported Endpoints
Randomized Controlled Trials	ProtecT Trial (Hamdy 2023) [25]	Phase 3 RCT (UK)	545	PSA 3.0–19.9, cT1c-T2, GG1–2	Pre-MRI Era (PSA/DRE only)	15.0	PCSS, OS, Metastases, Conversion
	SPCG-4 Trial (Bill-Axelson 2018) [26]	Randomized Trial (Scandinavia)	345	cT1b-T2, PSA < 50, GG1–2	Pre-MRI Era (Biopsy only)	23.2	PCSS, Overall Survival
	PIVOT Trial (Wilt 2017) [27]	Randomized Trial (USA)	367	cT1-T2, PSA ≤ 50, Age < 75	Pre-MRI Era (Biopsy only)	12.7	All-cause and PCSS Mortality
	ASIST Trial (Klotz 2019) [28]	Phase 2 Multicenter RCT	273	GG1–2, PSA ≤ 15, ≤50% cores	Baseline mpMRI vs. TRUS randomization	3.1	Upgrade-free survival at 2 yr
Prospective Cohorts & Registries	Canary PASS (Newcomb 2024) [29]	Prospective Multicenter (USA)	2155	GG1–2, PSA ≤ 20, ≤33% cores	Selective/Serial mpMRI	7.2	Reclassification, TFS, PCSS
	Johns Hopkins (Tosoian 2020) [35]	Prospective Institutional (USA)	1818	Epstein criteria (cT1c, PSA ≤ 10, PSAD < 0.15)	Protocol-driven mpMRI	7.6	PCSS, MFS, Upgrade-free survival
	MSKCC (Carlsson 2020) [36]	Retrospective Institutional (USA)	2664	Low & Favorable Intermediate-Risk	Baseline & Confirmatory mpMRI	5.8	10-yr MFS, PCSS, Deferred treatment
	Sunnybrook Cohort (Klotz 2015) [37]	Prospective Institutional (Canada)	993	PSA ≤ 10, GG1 (or GG2 if age > 70)	Selective mpMRI (Late adoption)	6.4	10-yr/15-yr PCSS, Conversion to RP
	Ontario Registry (Timilshina 2023) [32]	Population-Based Registry (Canada)	9311	Low & Intermediate-risk PCa	Registry-level practice (Variable)	8.4	10-yr PCSS, 10-yr OS
	PRIAS Study (Bokhorst 2016) [30]	International Prospective Registry	5302	cT1c-T2a, PSA ≤ 10, PSAD < 0.2, GG1	Serial biopsies +/− mpMRI	6.5	Discontinuation rate, Upgrading
	GÖTEBORG-1 (Palmstedt 2025) [8]	Randomized Screening Cohort (Sweden)	488	Screen-detected T1c-T2, PSA < 10	Late-adoption mpMRI	15.2	10-yr/15-yr PCSS, Conversion
	UCSF Cohort (Shee 2025) [38]	Prospective Cohort (USA)	774	cT1-T2, PSA ≤ 20, GG1–2	Standard longitudinal mpMRI	6.1	TFS, Upgrading to ≥GG3
Diagnostic Accuracy mpMRI Cohorts	PROMM-AS (Valentin 2024) [39]	Prospective Diagnostic Cohort (UK)	358	GG1 and low-volume GG2 on AS	Serial mpMRI + PRECISE Scoring	4.2	Diagnostic accuracy, Upgrade to ≥GG2
	UCLH MRI-AS (Stavrinides 2020) [40]	Prospective Imaging Cohort (UK)	472	GG1–2, Low-to-Intermediate Risk	Serial mpMRI + Targeted Biopsies	5.0	Progression-free survival, PRECISE
	Gallagher Cohort (2019) [41]	Longitudinal MRI Cohort (UK)	207	GG1–2 eligible for active surveillance	Longitudinal serial mpMRI	3.8	Sensitivity, Specificity of mpMRI
	Transatlantic (Sushentsev 2025) [42]	Transatlantic Multicenter Cohort	875	Baseline GG1–2, PI-RADS 1–5	Serial mpMRI with Cribriform Review	5.1	Cribriform pathology, PI-RADS failure
PROMs & Quality of Life Registries	PCO Registry (Al-Nader 2026) [44]	Prospective Multi-Registry (Global)	6265	Localized PCa (AS vs. RP vs. RT)	Standard Practice	2.0	PROMs (EPIC-26 Continence, Sexual, Bowel)
	SA-PCCOC Registry (Tiruye 2024) [33]	Population Clinical Registry (Australia)	8513	Biopsy-confirmed localized PCa	Standard Practice	5.0	PROMs (EPIC-26 Functional Recovery)
	NPCR Sweden (Corsini 2025) [34]	National Population Registry (Sweden)	3672	Screen/clinically detected localized PCa	Standard Practice	4.0	PROMs (EPIC-26 Quality of Life)

RCT = randomized controlled trial; AS = active surveillance; RP = radical prostatectomy; RT = radiotherapy; GG = Grade Group; PSA = prostate-specific antigen; PSAD = PSA density; mpMRI = multiparametric magnetic resonance imaging; PCSS = prostate cancer-specific survival; OS = overall survival; MFS = metastasis-free survival; TFS = treatment-free survival; PRECISE = Prostate Cancer Radiological Estimation of Change in Sequential Evaluation; PROMs = patient-reported outcome measures; EPIC-26 = Expanded Prostate Cancer Index Composite26-item.

**Table 2:** Quantitative meta-analytic synthesis of long-term oncologic survival, dynamic mpMRI diagnostic accuracy, and multivariable predictors of disease progression.

Analysis Category	Outcome/Endpoint Evaluated	Studies, n (Patients, N)	Pooled Summary [95% CI]	Statistical Model	Heterogeneity (I^2^)	*p*-Value
10-Year Oncologic Survival	Prostate Cancer-Specific Survival (PCSS)	10 (24,987)	98.9% [98.4–99.4%]	GLMM Random	38.4%	<0.001
	Metastasis-Free Survival (MFS)	8 (14,820)	96.2% [94.7–97.4%]	Random-effects	44.2%	<0.001
	Treatment-Free Survival (TFS/Non-conversion)	12 (18,450)	54.2% [48.1–60.3%] (95% PI: 31.4–75.8%)	Random-effects	88.1%	<0.001
mpMRI Diagnostic Performance	Sensitivity for Upgrading (≥GG2)	7 (2864)	85.6% [74.2–92.6%]	Reitsma Bivariate	62.0%	<0.001
	Specificity for Upgrading (≥GG2)	7 (2864)	56.2% [38.9–72.1%]	Reitsma Bivariate	71.4%	0.004
	Negative Predictive Value (PRECISE 1–3)	6 (2410)	93.4% [89.1–96.3%]	Random-effects	31.2%	<0.001
	Summary ROC Area Under Curve (AUC)	7 (2864)	0.84 [0.79–0.89]	Hierarchical sROC	N/A	<0.001
Multivariable Predictors (aHRs)	GG2 with Cribriform Pattern vs. Non-Cribriform	2 (1420)	12.67 [4.77–33.64]	Cox Random (aHR)	0.0%	<0.001
	mpMRI-Visible Target Lesion (PI-RADS 3–5 vs. 1–2)	4 (3120)	4.38 [1.84–10.44]	Cox Random (aHR)	34.1%	0.001
	Serial MRI Progression (PRECISE 4–5 vs. 1–3)	3 (1850)	2.80 [2.18–4.03]	Cox Random (aHR)	18.5%	<0.001
	Baseline PSA Density ≥ 0.15 ng/mL/cm3	5 (4210)	1.90 [1.10–3.29]	Cox Random (aHR)	41.0%	0.021
Subgroup & Era Analyses	Gleason Grade: GG1 vs. Low-Volume GG2 (PCSS)	6 (11,400)	99.8% vs. 98.2%	Subgroup GLMM	22.1%	0.082
	Gleason Grade: GG1 vs. Low-Volume GG2 (MFS)	4 (7850)	98.4% vs. 94.1%	Subgroup Random	28.4%	0.041
	Diagnostic Era: MRI-Era vs. Pre-MRI Era (MFS)	8 (14,820)	97.8% vs. 94.2%	Subgroup Random	35.0%	0.028
	Study Quality: Low RoB vs. Moderate RoB (PCSS)	10 (24,987)	99.1% vs. 98.4%	Subgroup GLMM	31.2%	0.210

CI = confidence interval; aHR = adjusted hazard ratio; GLMM = generalized linear mixed model; sROC = summary receiver operating characteristic; RoB = risk of bias; PRECISE = Prostate Cancer Radiological Estimation of Change in Sequential Evaluation. Statistical significance set at *p* < 0.05. Random-effects models calculated using restricted maximum likelihood (REML).

**Table 3:** GRADE (Grading of Recommendations Assessment, Development, and Evaluation) summary of findings and evidence certainty profile.

Clinical Endpoint/Outcome	Anticipated Absolute Effect [95% CI]	Patients, N (Studies, n)	Risk of Bias	Inconsistency	Indirectness	Imprecision	Certainty of Evidence (GRADE)	Clinical Interpretation & Summary Assessment
10-Year Prostate Cancer-Specific Survival (PCSS)	98.9% (95% CI: 98.4–99.4%)	24,987 (10 studies)	Low	Not serious (I^2^ = 38.4%)	Not serious	Not serious	HIGH	Active surveillance confers exceptional long-term oncologic safety in appropriately selected cohorts.
10-Year Metastasis-Free Survival (MFS)	96.2% (95% CI: 94.7–97.4%)	14,820 (8 studies)	Low	Not serious (I^2^ = 44.2%)	Not serious	Not serious	HIGH	Distant dissemination is rare; contemporary confirmatory imaging significantly minimizes metastatic progression.
Diagnostic NPV of Stable mpMRI (PRECISE 1–3)	93.4% (95% CI: 89.1–96.3%)	2410 (6 studies)	Low	Not serious (I^2^ = 31.2%)	Not serious	Serious (Trigger variance)	MODERATE	Serial radiologic stability (PRECISE 1–3) safely rules out high-grade upgrading, enabling biopsy deferral.
Progression Risk in GG2 with Cribriform Pattern	aHR: 12.67 (95% CI: 4.77–33.64)	1420 (2 studies)	Low	Not serious (I^2^ = 0.0%)	Not serious	Serious (Limited cohorts)	MODERATE	Invasive cribriform pattern represents a strong contraindication to AS; immediate active therapy indicated.
12-Month Urinary Continence Preservation (PROMs)	+3.0 pts change (No clinically meaningful decline vs. MID: −6 pts)	18,450 (3 registries)	Low	Not serious	Not serious	Not serious	HIGH	AS completely avoids post-treatment urinary incontinence, significantly outperforming RP (−17 pts).
12-Month Sexual Function Preservation (PROMs)	−2.0 pts change (No clinically meaningful decline vs. MID: −11 pts)	18,450 (3 registries)	Low	Not serious	Not serious	Not serious	HIGH	Baseline erectile function is maintained on AS, avoiding profound declines seen after RP (−35 pts) and RT (−12 pts).

GRADE Working Group grades of evidence: High certainty = further research is very unlikely to change our confidence in the estimate of effect; moderate certainty = further research is likely to have an important impact on our confidence and may change the estimate; low certainty = further research is very likely to have an important impact; very low certainty = any estimate of effect is very uncertain. CI = confidence interval; aHR = adjusted hazard ratio; NPV = negative predictive value; PRECISE = Prostate Cancer Radiological Estimation of Change in Sequential Evaluation; MID = Minimal Important Difference; RP = radical prostatectomy; RT = radiotherapy; PROMs = patient-reported outcome measures.

## Data Availability

The data supporting the findings of this study are available within the article and its Supplementary Materials. Further extraction sheets are available from the corresponding author upon reasonable request.

## Supplementary Materials

The following supporting information can be downloaded at https://www.mdpi.com/article/10.3390/cancers18203378/s1. Table S1: Detailed clinical trade-off and patient benefit matrix comparing active surveillance against immediate radical prostatectomy and external beam radiotherapy. Table S2: Diagnostic accuracy, clinical yield, and safety comparison of longitudinal surveillance modalities for detecting Grade Group ≥2 disease. Table S3: Detailed comparative analysis of active surveillance protocols, follow-up schedules, imaging integration, and triggers for radical intervention across major cohorts and RCTs. Table S4: Comprehensive PRISMA 2020 search strategy strings, electronic databases queried, interface hosts, search syntax, limits, and verified yields. Table S5: Univariable random-effects meta-regression and leave-one-out sensitivity analyses evaluating study-level moderators of survival and progression. Table S6: Longitudinal patient-reported functional trajectories (EPIC-26) across baseline, 12, 24, and 60 months of follow-up for active surveillance versus radical prostatectomy and radiotherapy. Figure S1: Methodological quality and risk of bias assessment panels across included studies (ROBINS-I for observational cohorts, Cochrane RoB 2 for randomized trials, and QUADAS-2 for diagnostic accuracy series).
