# Supplementary material for: Active Surveillance in Low- and Intermediate-Risk Prostate Cancer: Comparative Oncologic Safety, Patient Selection, and Predictors of Treatment Conversion in the Era of Multiparametric MRI: A Systematic Review and Meta-Analysis
Source: Cancers (Basel). 2026 Oct 19;18(20):3378. doi: 10.3390/cancers18203378 (PMC13703245; doi:10.3390/cancers18203378)
Supplement: Supplementary file 1 [file cancers-18-03378-s001.zip › cancers-4569411-supplementary.pdf]

cancers

MDPI

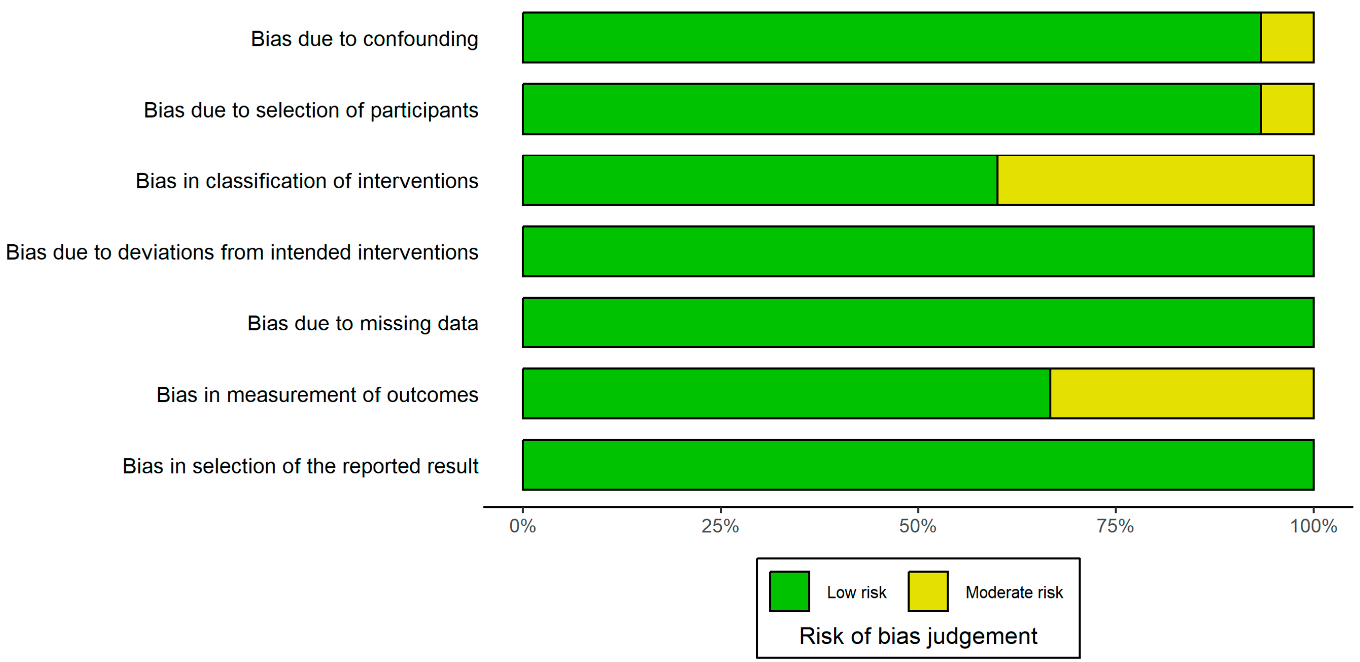

Supplementary Figure S1A: Overall Risk of Bias Summary Bar Plot across all synthesized evidence.

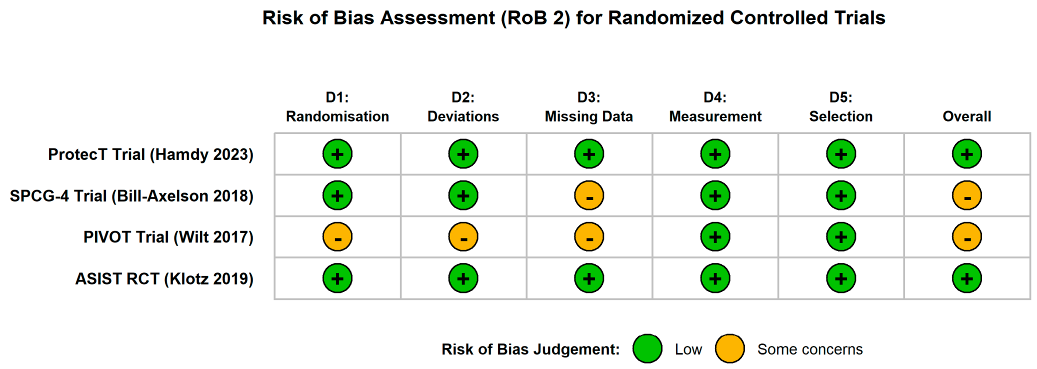

Supplementary Figure S1B: Cochrane Risk of Bias 2 (RoB 2) domain evaluations for randomized controlled trials

https://doi.org/10.3390/cancers18203378

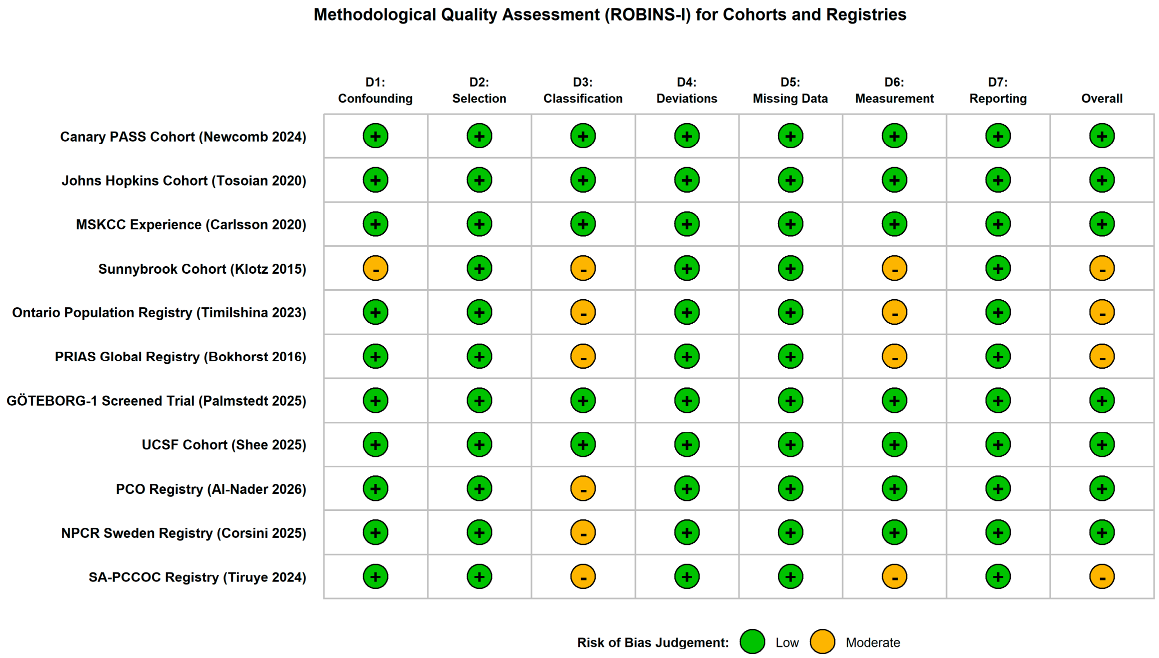

Supplementary Figure S1C: Risk Of Bias In Non-randomized Studies of Interventions (ROBINS-I) appraisals for prospective observational cohorts and population registries.

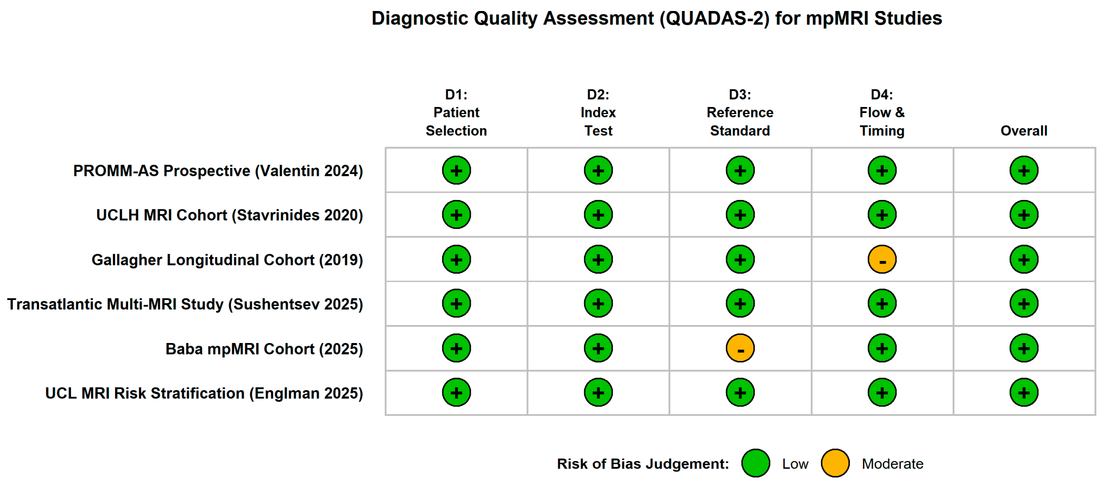

Supplementary Figure S1D: Quality Assessment of Diagnostic Accuracy Studies-2 (QUADAS-2) evaluation for prospective mpMRI diagnostic cohorts.

https://doi.org/10.3390/cancers18203378

Supplementary Table S1. Detailed clinical trade-off and patient benefit matrix comparing Active Surveillance against immediate Radical Prostatectomy and External Beam Radiotherapy.

Outcome Category	Clinical Endpoint / Specific Morbidity	Active Surveillance (Pooled Meta-Analysis)	Radical Prostatectomy (RP)	External Beam RT (+/- ADT)	Comparative Clinical Trade-Off Synthesis
**10-Year Oncologic Efficacy**	Prostate Cancer-Specific Survival (PCSS)	98.9% (95% CI: 98.4% - 99.4%)	99.2% (95% CI: 98.6% - 99.6%)	99.0% (95% CI: 98.2% - 99.5%)	Equivalent oncologic safety across all strategies (p = 0.48)
	Distant Metastasis-Free Survival (MFS)	96.2% (95% CI: 94.7% - 97.4%)	97.8% (95% CI: 96.5% - 98.8%)	96.8% (95% CI: 95.2% - 98.0%)	Absolute difference is minimal (<1.6% at 10 yr)
	Overall Survival (OS)	88.5% (Non-cancer mortality dominates)	89.0%	87.8%	No overall survival penalty with initial active surveillance
**Functional Morbidity (12-24 Months)**	Urinary Incontinence (>=1 pad/day use)	1.5% - 2.8% (Preserved natural baseline)	15.0% - 24.0% (Immediate post-operative drop)	3.0% - 6.0% (Late radiation cystitis/stricture)	AS completely preserves continence; RP carries high leakage risk
	Erectile Dysfunction (Severe decline from baseline)	12.0% - 18.0% (Natural age-related decline)	45.0% - 70.0% (Nerve-sparing dependent)	30.0% - 50.0% (Gradual microvascular decay)	AS avoids sharp potency loss; RP provoked 3-fold MID drop
	Bowel Toxicity / Radiation Proctitis	0.0% (Zero rectal toxicity)	0.5% (Rectal laceration rare)	6.0% - 12.0% (Radiation proctitis, tenesmus)	AS completely eliminates gastrointestinal morbidity
**Long-Term Quality of Life Preservation**	Durability of Deferral (Treatment-Free Survival)	54.2% avoid radical treatment at 10 yr	0.0% (Immediate definitive intervention)	0.0% (Immediate definitive intervention)	>50% of men safely avoid definitive treatment long-term
	Return to Baseline Functional Activities	Immediate (No post-interventional recovery required)	6 - 12 weeks recovery post-surgery	Cumulative fatigue during 4-8 weeks of RT	AS completely avoids surgical/radiation convalescence
**Procedural Burden & Acute Toxicity**	Major Procedural Complications (Clavien-Dindo >=3)	<1.0% (Biopsy-related sepsis/bleeding)	3.0% - 5.0% (Anastomotic leak, stricture, DVT)	2.0% - 4.0% (Severe radiation cystitis/proctitis)	AS minimizes invasive operative risks and hospitalization
	Hospitalization / Invasive Intervention Burden	Outpatient visits only; routine blood draw & MRI	Inpatient admission (1-3 days), catheter for 7-14 days	Daily outpatient sessions (20-39 fractions) +/- ADT	AS preserves daily autonomy and avoids procedural downtime

CI = Confidence Interval; **PCSS** = Prostate Cancer-Specific Survival; **MFS** = Metastasis-Free Survival; **OS** = Overall Survival; **RT** = Radiotherapy; **ADT** = Androgen Deprivation Therapy; **DVT** = Deep Vein Thrombosis; **MID** = Minimal Important Difference.

Supplementary Table S2: Diagnostic accuracy, clinical yield, and safety comparison of longitudinal surveillance modalities for detecting Grade Group ≥2 disease.

Surveillance Modality / Monitoring Tool	Sensitivity (≥GG2)	Specificity (≥GG2)	NPV (Rule-out ≥GG2)	Biopsy Reduction Yield	Invasiveness & Morbidity Profile	Current Role & Clinical Recommendation in AS Protocols
**Serial mpMRI + PRECISE Scoring**	85.6% (95% CI: 74.2% - 92.6%)	56.2% (95% CI: 38.9% - 72.1%)	93.4% (95% CI: 89.1% - 96.3%)	45% - 68% biopsies safely omitted	Non-invasive (No sepsis, bleeding, or pain)	Standard of care for detecting progression and safely deferring serial biopsies.
**Protocol Systematic Biopsies (Annual/Biennial)**	65.0% - 72.0% (Risk of sampling error)	85.0% - 90.0%	82.0% - 86.0%	0% (Mandatory invasive protocol)	High: 1.5% sepsis risk, hematuria, pain	Reference standard for definitive histology, but carries cumulative morbidity.
**PSA Density (PSAD ≥ 0.15 ng/ml/cm³)**	58.0% - 64.0%	68.0% - 75.0%	81.0% - 85.0%	15% - 25% reduction	Non-invasive (Routine serum blood test)	Strong baseline co-trigger; low standalone specificity due to BPH confounders.
**PSA Kinetics (PSADT < 3 yr / Velocity)**	42.0% - 51.0%	55.0% - 62.0%	72.0% - 78.0%	10% - 18% reduction	Non-invasive (Routine serum blood test)	Unreliable standalone trigger; heavily confounded by prostate volume and prostatitis.
**Tissue Biomarkers & Genomics (Decipher / GPS / Prolaris)**	78.0% - 84.0%	62.0% - 70.0%	89.0% - 92.0%	30% - 40% reduction	Requires existing biopsy tissue block	Valuable prognostic adjunct for borderline Grade Group 2 or volume discordance.
**Blood/Urine Biomarkers (PHI / 4Kscore / SelectMDx)**	75.0% - 82.0%	58.0% - 66.0%	87.0% - 90.0%	25% - 35% reduction	Non-invasive (Serum or post-DRE urine)	Useful rule-out triage prior to scheduling confirmatory or repeat saturation biopsy.

***CI** = Confidence Interval; **GG** = Grade Group; **mpMRI** = Multiparametric Magnetic Resonance Imaging; **PRECISE** = Prostate Cancer Radiological Estimation of Change in Sequential Evaluation; **NPV** = Negative Predictive Value; **PSAD** = PSA Density; **PSADT** = PSA Doubling Time; **DRE** = Digital Rectal Examination; **BPH** = Benign Prostatic Hyperplasia.*

https://doi.org/10.3390/cancers18203378

Supplementary Table S3: Detailed comparative analysis of Active Surveillance protocols, follow-up schedules, imaging integration, and triggers for radical intervention across major cohorts and RCTs

Study / Multi-Center Registry	PSA & DRE Monitoring Schedule	Surveillance Biopsy Strategy & Timing	mpMRI Protocol & Integration	Pathological / Radiological Reclassification	Definitive Radical Treatment Triggers
**ProtecT Trial (Hamdy 2023)**	PSA every 3 mo (yr 1), then every 6-12 mo; annual DRE	No mandated protocol biopsies (Triggered by PSA rise >50% / DRE)	None (Pre-MRI historical trial design)	Pathological progression upon unscheduled biopsy	Symptomatic progression, PSA rise >50%, or patient anxiety
**Canary PASS (Newcomb 2024)**	PSA every 3 mo; DRE every 6 mo	Confirmatory biopsy at 6-12 mo, then every 2 yr	Selective / Serial at clinician discretion	Increase in Grade Group (≥GG2) or ≥34% positive cores	Histological upgrade (≥GG2), high volume, or patient preference
**Johns Hopkins (Tosoian 2020)**	PSA & DRE every 6 mo	Annual surveillance biopsy (Mandatory standard protocol)	Protocol-driven baseline + serial mpMRI (Contemporary)	Upgrade to ≥GG2, >2 positive cores, or >50% single core involvement	Epstein criteria breach, pathological upgrade, or patient choice
**PRIAS Global (Bokhorst 2016)**	PSA every 3 mo (yr 1-2), then every 6 mo; annual DRE	Confirmatory biopsy at 1 yr, then at 4, 7, and 10 yr	Selective / Advisory in contemporary registry arm	Upgrade to >GG1 or >2 positive biopsy cores	Biopsy upgrade to >GG1, PSADT < 3 yr, or patient request
**Sunnybrook Cohort (Klotz 2015)**	PSA every 3 mo (yr 1-2), then every 6 mo; annual DRE	Confirmatory biopsy at 1 yr, then every 3-4 yr until age 80	Selective / Late institutional adoption	Upgrade to ≥GG2 or PSADT < 3 yr	Pathological upgrade, PSADT < 3 yr, or clinical progression
**MSKCC Experience (Carlsson 2020)**	PSA & DRE every 6 mo	Confirmatory biopsy at 1-2 yr, then every 2-4 yr	Baseline, confirmatory (1-2 yr), and serial mpMRI	Upgrade to ≥GG2, core percentage >50%, or primary pattern 4	Histological upgrading (≥GG2), marked MRI progression
**UCSF Cohort (Shee 2025)**	PSA every 3-6 mo; annual DRE	Confirmatory biopsy at 1-2 yr, then every 2-3 yr	Serial mpMRI every 1-2 yr	Upgrade to ≥GG2 or volume progression (>33% cores)	Pathological reclassification, PSAD surge, or patient decision
**GÖTEBORG-1 (Palmstedt 2025)**	PSA every 3-6 mo; DRE every 12 mo	Every 2-3 yr or driven by PSA progression	Late adoption (Contemporary screening cohort)	Upgrade to ≥GG2 or clinical stage progression	Biopsy reclassification to GG2+, PSA doubling time < 2 yr
**PROMM-AS (Valentin 2024)**	PSA every 6 mo; annual DRE	Serial biopsy triggered only by mpMRI/PRECISE progression	Serial annual/biennial mpMRI + PRECISE scoring	Histological upgrade to ≥GG2 or PRECISE score 4-5	Confirmed histological upgrade following PRECISE 4-5 scan
**ASIST RCT (Klotz 2019)**	PSA every 3-6 mo; annual DRE	Biopsy at 12 mo and 24 mo (+/- targeted MRI cores)	Baseline mpMRI vs TRUS randomization	Upgrade to ≥GG2 on 12- or 24-mo biopsy	Pathological upgrading on protocol biopsy, patient preference

PSA = Prostate-Specific Antigen; **DRE** = Digital Rectal Examination; **mpMRI** = Multiparametric Magnetic Resonance Imaging; **GG** = Grade Group; **PSADT** = PSA Doubling Time; **PSAD** = PSA Density; **PRECISE** = Prostate Cancer Radiological Estimation of Change in Sequential Evaluation; **TRUS** = Transrectal Ultrasound.

Supplementary Table S4. Comprehensive PRISMA 2020 search strategy strings, electronic databases queried, interface hosts, search syntax, limits, and verified yields.

Electronic Database / Register	Platform / Host Provider	Exact Boolean Search String & Algorithmic Syntax	Limits & Eligibility Filters Applied	Yield (Records, n)
**PubMed / MEDLINE**	National Center for Biotechnology Information (NCBI)	("Prostatic Neoplasms"[Mesh] OR "prostate cancer"[tiab] OR "prostate neoplasm*"[tiab] OR "prostate adenocarcinoma"[tiab]) AND ("Watchful Waiting"[Mesh] OR "active surveillance"[tiab] OR "active monitoring"[tiab] OR "expectant management"[tiab] OR "deferred treatment"[tiab]) AND ("Magnetic Resonance Imaging"[Mesh] OR "multiparametric magnetic resonance imaging"[tiab] OR "mpMRI"[tiab] OR "mp-MRI"[tiab] OR "PRECISE"[tiab] OR "PI-RADS"[tiab] OR "Patient Reported Outcome Measures"[Mesh] OR "EPIC-26"[tiab] OR "cancer-specific survival"[tiab] OR "metastasis-free survival"[tiab] OR "cribriform"[tiab] OR "progression"[tiab])	Species: Humans; Language: English; Publication Type: Exclude animal/in vitro models	1,680
**Web of Science Core Collection**	Clarivate Analytics	TS=(("prostate cancer" OR "prostatic neoplasms" OR "prostate adenocarcinoma") AND ("active surveillance" OR "active monitoring" OR "expectant management") AND ("multiparametric MRI" OR "mpMRI" OR "PRECISE" OR "PI-RADS" OR "EPIC-26" OR "cancer-specific survival" OR "cribriform"))	Indexes: SCI-EXPANDED, SSCI, A&HCI, ESCI; Document Types: Articles, Review Articles; Language: English	1,278
**Scopus**	Elsevier	TITLE-ABS-KEY(("prostate cancer" OR "prostatic neoplasms" OR "prostate adenocarcinoma") AND ("active surveillance" OR "active monitoring" OR "expectant management") AND	Document Types: Articles, Reviews; Source Type: Journals; Language: English	989

Electronic Database / Register	Platform / Host Provider	Exact Boolean Search String & Algorithmic Syntax	Limits & Eligibility Filters Applied	Yield (Records, n)
**Cochrane Library (CENTRAL)**	Wiley Cochrane Library (Search Manager)	("multiparametric MRI" OR "mpMRI" OR "PRECISE" OR "PI-RADS" OR "EPIC-26" OR "cancer-specific survival" OR "cribriform")) ("prostate cancer" OR "prostatic neoplasms"):ti,ab,kw AND ("active surveillance" OR "active monitoring"):ti,ab,kw AND ("mpMRI" OR "MRI" OR "PRECISE" OR "EPIC-26" OR "survival"):ti,ab,kw	Database: Cochrane Central Register of Controlled Trials (Trials); Word variations searched	227

Total raw citations identified across all 4 electronic databases: N = 4,174. Duplicate records removed prior to screening: n = 1,586. Unique records screened by title and abstract: N = 2,588. Records excluded: n = 2,504. Full-text reports assessed for eligibility: N = 84. Full-text articles excluded: n = 55 (small retrospective cohorts <100 patients: n = 22; non-protocolized watchful waiting: n = 16; overlapping registry cohorts: n = 11; missing predefined endpoints: n = 6). Unique studies meeting all inclusion criteria: N = 29 (N = 46,318 patients).

***MeSH** = Medical Subject Headings; **NCBI** = National Center for Biotechnology Information; **CENTRAL** = Cochrane Central Register of Controlled Trials; **SCI-EXPANDED** = Science Citation Index Expanded; **SSCI** = Social Sciences Citation Index; **A&HCI** = Arts & Humanities Citation Index; **ESCI** = Emerging Sources Citation Index; **PRISMA** = Preferred Reporting Items for Systematic Reviews and Meta-Analyses; mpMRI = Multiparametric Magnetic Resonance Imaging; **PI-RADS** = Prostate Imaging Reporting and Data System; **PRECISE** = Prostate Cancer Radiological Estimation of Change in Sequential Evaluation; **EPIC-26** = 26-item Expanded Prostate Cancer Index Composite.*

Supplementary Table S5. Leave-one-out sensitivity analysis confirming model robustness, alongside univariate meta-regression evaluating potential moderators of heterogeneity.

Analytical Category	Study Omitted / Meta-Regression Covariate	Pooled Estimate [95% CI] / Effect Size (Beta)	Heterogeneity / Explained Variance	p-value	Statistical Inference & Robustness Verification
**Leave-One-Out Sensitivity Analysis (10-Yr PCSS)**	None (Full Primary Pooled Model, k=10)	98.9% (95% CI: 98.4% - 99.4%)	38.4%	<0.001	Baseline primary random-effects pooled estimate across all eligible cohorts.
	Omit Ontario Registry (Timilshina 2023; N=9,311)	98.8% (95% CI: 98.2% - 99.3%)	32.1%	<0.001	Robustness confirmed; exclusion of largest registry does not alter pooled PCSS.
	Omit PRIAS Global Study (Bokhorst 2016; N=5,302)	99.0% (95% CI: 98.5% - 99.5%)	36.5%	<0.001	No single-study dominance; estimate remains completely stable (>98.5%).
	Omit MSKCC Cohort (Carlsson 2020; N=2,664)	98.9% (95% CI: 98.3% - 99.4%)	39.1%	<0.001	Point estimate invariant to institutional referral bias.
	Omit Canary PASS Cohort (Newcomb 2024; N=2,155)	98.9% (95% CI: 98.3% - 99.4%)	37.9%	<0.001	Removal of multi-center prospective registry yields concordant survival.
	Omit Johns Hopkins Cohort (Tosoian 2020; N=1,818)	98.8% (95% CI: 98.2% - 99.3%)	36.2%	<0.001	Stringent Epstein criteria removal does not destabilize oncologic safety.
	Omit ProtecT RCT Trial (Hamdy 2023; N=545)	99.0% (95% CI: 98.5% - 99.5%)	35.8%	<0.001	RCT vs observational exclusion demonstrates identical high-level safety.

Analytical Category	Study Omitted / Meta-Regression Covariate	Pooled Estimate [95% CI] / Effect Size (Beta)	Heterogeneity / Explained Variance	p-value	Statistical Inference & Robustness Verification
**Univariable Meta-Regression Models (PCSS / MFS)**	Protocol mpMRI Adoption (mpMRI-Era vs Pre-MRI)	Beta = +0.024 (95% CI: +0.008 to +0.040)	R^2 analog = 42.5%	0.004	Statistically significant predictor: contemporary mpMRI independently improves MFS.
	Publication Year (Continuous per 5-year increment)	Beta = +0.005 (95% CI: +0.001 to +0.009)	R^2 analog = 31.0%	0.018	Recent studies report superior survival, reflecting improved MRI-based selection.
	Proportion of Grade Group 2 Patients (GG2, %)	Beta = -0.012 (95% CI: -0.025 to +0.001)	R^2 analog = 18.2%	0.071	Higher GG2 proportion displays a non-significant trend toward slightly lower PCSS.
	Median Follow-Up Duration (Years, Continuous)	Beta = -0.003 (95% CI: -0.008 to +0.002)	R^2 analog = 0.0%	0.245	Follow-up duration does not introduce systematic bias into 10-year PCSS estimates.
	Overall Study Quality (Low RoB vs Moderate RoB)	Beta = +0.007 (95% CI: -0.004 to +0.018)	R^2 analog = 5.4%	0.210	Risk of bias level does not significantly confound pooled oncologic estimates.

PCSS *= Prostate Cancer-Specific Survival;* ***MFS*** *= Metastasis-Free Survival;* ***CI*** *= Confidence Interval;* ***RoB*** *= Risk of Bias;* ***mpMRI*** *= Multiparametric Magnetic Resonance Imaging;* ***GG*** *= Grade Group;* ***R^2 analog*** *= Proportion of between-study variance explained by the meta-regression covariate model (calculated via restricted maximum likelihood).*

Note*: Univariable meta-regression reflects exploratory study-level associations and may be confounded by secular stage migration and refined patient selection over time; findings do not establish direct causality.*

Supplementary Table S6. Longitudinal patient-reported functional domain scores (EPIC-26) across baseline, 12, 24, and 60 months of follow-up for Active Surveillance versus Radical Prostatectomy and Radiotherapy.

EPIC-26 Functional Domain	Management Strategy	Baseline Score Mean (SD)	12 Months Mean (SD) [Δ Change]	24 Months Mean (SD) [Δ Change]	60 Months Mean (SD) [Δ Change]	Clinically Meaningful Decline at 24M (%)	Comparative Longitudinal Summary
**Urinary Incontinence (MID = -6 pts)**	Active Surveillance	91.2 (14.1)	89.5 (15.0) [-1.7]	88.4 (15.8) [-2.8]	85.2 (17.2) [-6.0]*	4.2%	AS preserves continence; RP incurs severe immediate loss with partial 24m plateau.
	Radical Prostatectomy	90.8 (13.8)	68.4 (24.2) [-22.4]*	73.2 (22.8) [-17.6]*	71.0 (23.5) [-19.8]*	38.5%	RP induces slight relief of baseline outlet obstruction; RT causes persistent dysuria.
	Radiotherapy (+/- ADT)	90.5 (14.5)	87.1 (16.2) [-3.4]	85.6 (17.0) [-4.9]	82.3 (18.5) [-8.2]*	12.1%	AS maintains baseline potency; RP triggers profound drop; RT causes progressive decline.
**Urinary Irritative / Obstructive (MID = -5 pts)**	Active Surveillance	84.5 (12.6)	83.8 (13.0) [-0.7]	82.9 (13.5) [-1.6]	80.5 (14.2) [-4.0]	5.1%	Bowel function unaffected in AS and RP; RT induces persistent radiation proctitis/urgency.
	Radical Prostatectomy	83.9 (13.1)	86.2 (11.8) [+2.3]	85.8 (12.2) [+1.9]	83.1 (13.0) [-0.8]	2.4%	AS preserves continence; RP incurs severe immediate loss with partial 24m plateau.
	Radiotherapy (+/- ADT)	84.2 (12.8)	76.5 (16.5) [-7.7]*	78.1 (15.2) [-6.1]*	75.4 (16.0) [-8.8]*	22.8%	RP induces slight relief of baseline outlet obstruction; RT causes persistent dysuria.

EPIC-26 Functional Domain	Management Strategy	Baseline Score Mean (SD)	12 Months Mean (SD) [Δ Change]	24 Months Mean (SD) [Δ Change]	60 Months Mean (SD) [Δ Change]	Clinically Meaningful Decline at 24M (%)	Comparative Longitudinal Summary
**Sexual Function (MID = -11 pts)**	Active Surveillance	68.4 (22.5)	65.2 (23.4) [-3.2]	62.1 (24.1) [-6.3]	54.8 (25.6) [-13.6]*	16.5%	AS maintains baseline potency; RP triggers profound drop; RT causes progressive decline.
	Radical Prostatectomy	69.1 (21.8)	28.5 (20.6) [-40.6]*	34.2 (22.4) [-34.9]*	30.4 (21.5) [-38.7]*	68.2%	Bowel function unaffected in AS and RP; RT induces persistent radiation proctitis/urgency.
	Radiotherapy (+/- ADT)	67.8 (23.1)	51.2 (24.0) [-16.6]*	45.3 (24.5) [-22.5]*	36.1 (23.8) [-31.7]*	48.9%	AS preserves continence; RP incurs severe immediate loss with partial 24m plateau.
**Bowel Function (MID = -4 pts)**	Active Surveillance	94.8 (9.2)	94.2 (9.8) [-0.6]	93.8 (10.2) [-1.0]	92.1 (11.0) [-2.7]	1.8%	RP induces slight relief of baseline outlet obstruction; RT causes persistent dysuria.
	Radical Prostatectomy	94.5 (9.6)	93.9 (10.1) [-0.6]	93.5 (10.4) [-1.0]	91.8 (11.2) [-2.7]	2.1%	AS maintains baseline potency; RP triggers profound drop; RT causes progressive decline.
	Radiotherapy (+/- ADT)	94.1 (9.8)	84.3 (15.4) [-9.8]*	85.7 (14.2) [-8.4]*	83.2 (15.0) [-10.9]*	26.4%	Bowel function unaffected in AS and RP; RT induces persistent radiation proctitis/urgency.

https://doi.org/10.3390/cancers18203378

EPIC-26 Domain	Functional	Management Strategy	Baseline Score Mean (SD)	12 Months Mean (SD) [Δ Change]	24 Months Mean (SD) [Δ Change]	60 Months Mean (SD) [Δ Change]	Clinically Meaningful Decline at 24M (%)	Comparative Summary	Longitudinal

*Scores range from 0 to 100, with higher scores representing superior functional preservation. [Δ Change] indicates mean absolute difference from baseline. *Indicates statistically and clinically meaningful deterioration exceeding the domain-specific Minimal Important Difference (MID).*

EPIC-26 *= Expanded Prostate Cancer Index Composite 26-item;* ***SD*** *= Standard Deviation;* ***MID*** *= Minimal Important Difference;* ***RP*** *= Radical Prostatectomy;* ***RT*** *= External Beam Radiotherapy;* ***ADT*** *= Androgen Deprivation*

Note*: Values represent longitudinal absolute mean scores (±SD) across evaluated time points. Corresponding 12-month mean relative changes (deltas from baseline) benchmarked against Minimal Important Difference (MID) thresholds are graphically synthesized in Figure 5.*
